# MCPdb: The bacterial microcompartment database

**DOI:** 10.1371/journal.pone.0248269

**Published:** 2021-03-29

**Authors:** Jessica M. Ochoa, Kaylie Bair, Thomas Holton, Thomas A. Bobik, Todd O. Yeates

**Affiliations:** 1 UCLA Molecular Biology Institute, University of California Los Angeles, Los Angeles, California, United States of America; 2 UCLA-DOE Institute for Genomics and Proteomics, University of California Los Angeles, Los Angeles, California, United States of America; 3 UCLA Department of Chemistry and Biochemistry, University of California Los Angeles, Los Angeles, California, United States of America; 4 Roy J. Carver Department of Biochemistry, Biophysics and Molecular Biology, Iowa State University, Ames, Iowa, United States of America; University of Nebraska-Lincoln, UNITED STATES

## Abstract

Bacterial microcompartments are organelle-like structures composed entirely of proteins. They have evolved to carry out several distinct and specialized metabolic functions in a wide variety of bacteria. Their outer shell is constructed from thousands of tessellating protein subunits, encapsulating enzymes that carry out the internal metabolic reactions. The shell proteins are varied, with single, tandem and permuted versions of the PF00936 protein family domain comprising the primary structural component of their polyhedral architecture, which is reminiscent of a viral capsid. While considerable amounts of structural and biophysical data have been generated in the last 15 years, the existing functionalities of current resources have limited our ability to rapidly understand the functional and structural properties of microcompartments (MCPs) and their diversity. In order to make the remarkable structural features of bacterial microcompartments accessible to a broad community of scientists and non-specialists, we developed MCPdb: The Bacterial Microcompartment Database (https://mcpdb.mbi.ucla.edu/). MCPdb is a comprehensive resource that categorizes and organizes known microcompartment protein structures and their larger assemblies. To emphasize the critical roles symmetric assembly and architecture play in microcompartment function, each structure in the MCPdb is validated and annotated with respect to: (1) its predicted natural assembly state (2) tertiary structure and topology and (3) the metabolic compartment type from which it derives. The current database includes 163 structures and is available to the public with the anticipation that it will serve as a growing resource for scientists interested in understanding protein-based metabolic organelles in bacteria.

## Introduction

Bacterial microcompartments (MCPs or alternatively BMCs), are supramolecular structures found in approximately 20% of bacteria across numerous phyla [1, 2]. These giant protein-based structures have evolved to serve organelle-like functions, with different MCP types encapsulating distinct enzymes in order to carry out specific metabolic processes in a sequestered environment within the cell interior [3–6]. MCPs are known to carry out diverse metabolic processes; their unifying functional feature is that they provide a mechanism for bacteria to perform certain multistep reactions in a way that retains metabolic intermediates inside the MCP. The co-localization of sequentially acting enzymes housed inside the MCP helps optimize metabolic flux while limiting alternative side reactions. Importantly, MCPs help prevent the efflux of toxic and/or volatile intermediates into the cytosol [3, 7, 8]. Bacterial microcompartments can be broadly classified into two major categories: carboxysomes and metabolosomes. Carboxysomes are the founding members of the MCPs. They enhance CO_2_ fixation in bacteria by encapsulating two sequentially acting enzymes–carbonic anhydrase and ribulose-1,5-bisphosphate carboxylase/oxygenase (RuBisCO) [4, 9, 10]. Bicarbonate (in addition to ribulose-bisphosphate) is the substrate that enters the carboxysome via diffusion across the shell; CO_2_ is the key intermediate, which is produced by carbonic anhydrase and must be consumed by RuBisCO prior to escape. By contrast, metabolosomes use an assortment of key enzymes to metabolize a variety of substrates including 1,2-propanediol for the propanediol utilization (PDU) MCP and ethanolamine for the ethanolamine utilization (EUT) [4, 7, 8, 10]. Other microcompartments utilize glycyl-radical chemistry (GRM MCPs) and can be further divided into subclasses based on their substrates and signature enzymes, including the glycyl-radical propanediol (Grp) MCP, the choline utilization (Cut) MCP and an additional GRM type that utilizes fucose and rhamnose [11–15]. Lastly, there are MCPs that have been more recently discovered whose metabolic functions are still emerging, including the RMM/Aaum MCP and the Etu MCP. Several recent structures of both BMC and BMV (bacterial microcompartment vertex) proteins have been determined for an MCP first called RMM (for *Rhodococcus and* *M**ycolicibacterium* *M**icrocompartment)* and then renamed Aaum (for its apparent role in amino acetone utilization [1, 5, 14, 16, 17]. Additionally, the Etu MCP, or the ethanol utilization microcompartment, has been observed in *Clostridium kluyveri* and has had one of its shell proteins characterized [18, 19].

**Fig 1:**
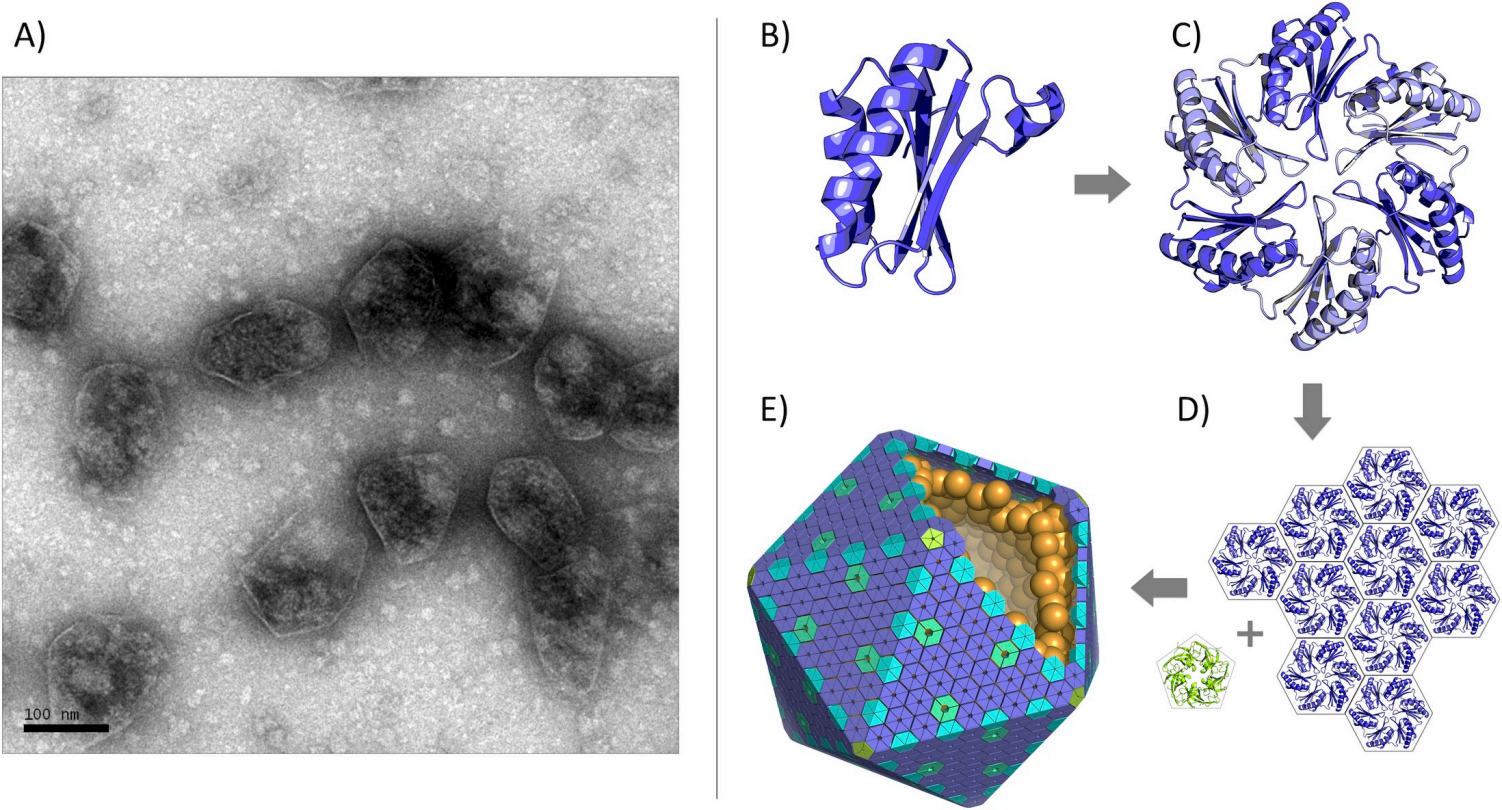
Bacterial microcompartments (MCPs) are large proteinaceous assemblies that function as metabolic organelles. (A) Negative stain electron micrograph of purified Pdu MCPs (scale bar: 50 nm). MCP shells are assembled primarily from proteins belonging to the BMC family (B), which are hexameric or trimeric pseudohexamers (C). (D) Hexameric and pseudohexameric BMC shell proteins pack laterally to form the facets while pentameric BMV proteins (lime green) of unrelated structure form the vertices (D). (E) An idealized model of a microcompartment with external shell proteins and encapsulated enzymes. Most natural MCP shells are not as geometrically regular as depicted here by the icosahedral architecture.

**Fig 2:**
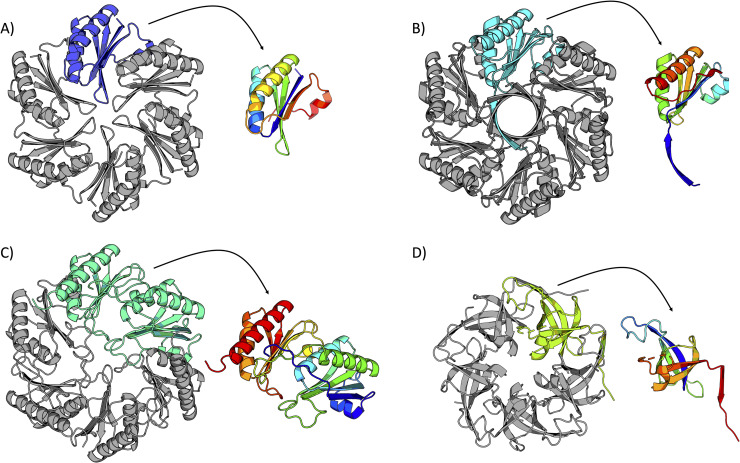
Cartoon representations of four bacterial microcompartment shell proteins. A single monomer is highlighted and presented in the context of the biological assembly, with a color-ramped (blue = N-terminus; red = C-terminus) version of the monomer adjacent to each structure. (A) A representative hexameric BMC shell protein (BMC-H) (PDB 2EWH) [24]. (B) A representative permuted BMC shell protein (PDB 6XPI) [31]. (C) A representative trimeric BMC shell protein (BMC-T) (PDB 3I82) [28]. (D) A representative BMV shell protein (PDB 4I7A) [15].

Despite their functional diversity, bacterial microcompartments are now understood to be structurally similar. Constructed entirely of proteins, the outer microcompartment shell is composed of thousands of homologous tessellating shell proteins belonging to the BMC protein family [20–22], whose structures were first elucidated in 2005 [23, 24]. The canonical BMC protein domain (Fig 1) oligomerizes to form hexameric disks with central pores for the (presumably) diffusive influx of metabolic substrates and the efflux of products. The hexameric disks pack laterally to form the nearly flat facets of the intact shell, while pentameric BMV proteins form the vertices of these large, polyhedral structures (Fig 1) [15, 20]. Any single microcompartment type is composed of multiple paralogs of the BMC protein, with different paralogs offering distinct structural properties. This roughly 100-amino acid domain (Pfam PF00936) remains the primary key for exploring and discovering new types of microcompartments, and has been extensively studied and characterized [15, 21, 22, 25–32]. Structural studies have revealed major topologically distinct variations of the BMC protein domain. The canonical form is the BMC-H shell protein; it is the most abundant, contains a single BMC domain and forms a cyclic homohexamer (Fig 2A) [23]. An alternate topological form of lesser-understood function occurs in the form of permuted BMC proteins [29]. These contain a single, essentially intact BMC domain with a circular permutation. This circular permutation results in a reordering of the amino acid sequence but a similar overall BMC protein fold (Fig 2B), with some of these structures revealing a high degree of flexibility and symmetry-breaking [28, 31]. The BMC-T (*T* stands for tandem) category of proteins consists of two tandem repeats of the BMC domain. BMC-Ts are cyclic trimers that form pseudohexamers (Fig 2C) whose overall shape closely resemble a canonical BMC hexamer [28, 33, 34]. Further variations exist within the BMC-T type, with some also exhibiting circular permutations. In some cases, BMC-T shell proteins have been shown to undergo large conformational changes between closed and open pore states, with critical implications for regulated transport [28, 33–36]. Moreover, some BMC-Ts and even some BMC-H shell proteins have been found to bind iron-sulfur clusters in their central pores [12, 30, 37]. Finally, BMV proteins (sometimes referred to as BMC-P) are cyclic homopentamers that form the vertices of bacterial microcompartments (Fig 2D) [15, 20, 38, 39]. These are based on the Pfam03319 protein domain, which is entirely unrelated in sequence and structure from the BMC protein domain family. The sophisticated mechanistic features of MCPs emphasize their qualification as true organelles in bacteria, built from proteins rather than a lipid bilayer.

**Fig 3:**
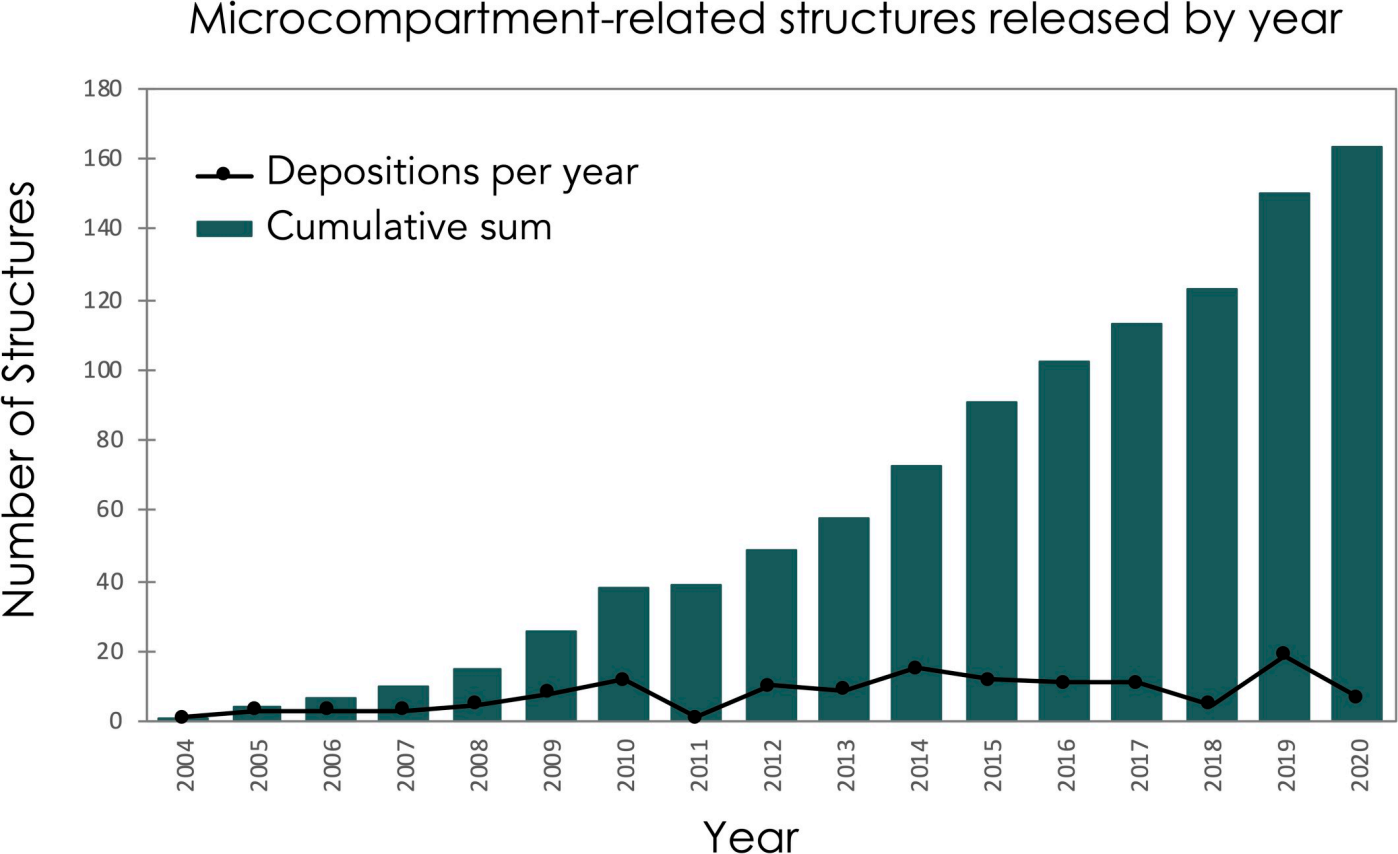
Growth over time of known microcompartment-related structures. There are currently 163 microcompartment and encapsulin-related protein structures deposited in the PDB. Structures were identified by using “microcompartment,” “carboxysome” and “encapsulin” as search terms in the PDB. The amino acid sequence of a few representative BMCs and BMVs were also used to ensure we identified all microcompartment shell proteins that have been deposited in the PDB.

Notwithstanding their wide distribution and the extensive investigation into their outer shells, microcompartments remain only partially understood. To date, more than 150 bacterial microcompartment-related structures have been characterized and deposited in the Protein Data Bank (PDB) (Fig 3). Various items of information about each structure–organism, amino acid sequence, functional name, etc.–are generally available, but other critical insights about structure and function are difficult to sort out from the raw data as it is typically presented, and this challenge is especially true for non-experts that have minimal familiarity with the PDB protein structure database. Because understanding quaternary structure–i.e. protein assembly states–is especially critical to understanding elements of MCP function, we viewed the challenges associated with identifying natural assembly forms as a major barrier for novices trying to generate and understand the natural biological forms of MCP shell proteins. We have also addressed MCP-specific aspects of form and function that are not easily discerned from raw structure files. Discrimination of diverse topological forms of BMC proteins is also provided. This is usually non-obvious from raw structural data files, and these structural variations often relate to important properties of the pores (e.g. ‘open’ or ‘closed’), which are routes for metabolite transport.

A distinct class of prokaryotic nanocompartments, known as encapsulins, has also come under recent investigation. Like MCPs, encapsulins are protein-based compartments from diverse prokaryotes that facilitate compartmentalization and cellular organization [40, 41]. They are icosahedral shells between 25–42 nm in diameter and capable of encapsulating one or more cargo proteins [40–42]. Encapsulin proteins are distinct from the BMC and BMV proteins of MCPs; structural similarity indicates that the encapsulin protein shares a common ancestor with the capsid proteins from the HK97 family of viruses [41]. Encapsulin shells generally require only a single protomer, which self-assembles to form the outer shell [41, 43, 44]. A growing body of research has demonstrated that encapsulins are capable of mitigating oxidative stress and functioning as iron storage containers [41, 43–46].

A growing appreciation of the uniqueness and biological importance of MCPs and other nanocompartments, an expanding body of data on their shell proteins, and current paucity of systematic annotation, motivated the development of a centralized database to address these knowledge gaps. Making bacterial microcompartments more accessible to not only structural experts but to a broader scientific audience should help advance this growing field of biology. Here, we describe the development of a novel database, MCPdb: The Bacterial Microcompartment Database (https://mcpdb.mbi.ucla.edu/). While metabolic compartments based on the common BMC protein architecture are the main focus of this database, we also make connections to other systems by including structural information on encapsulins. We collected all known bacterial microcompartment protein structures and assembled a novel online tool that provides users with simplified searching capabilities, structural and biophysical annotations and multiple visualization avenues for examining microcompartment biological assemblies. Most importantly, all structures in MCPdb have been validated–that is to say, quaternary structures have been manually confirmed using human-expertise-based curation.

## Materials and methods

### Data collection and curation

**Fig 4:**
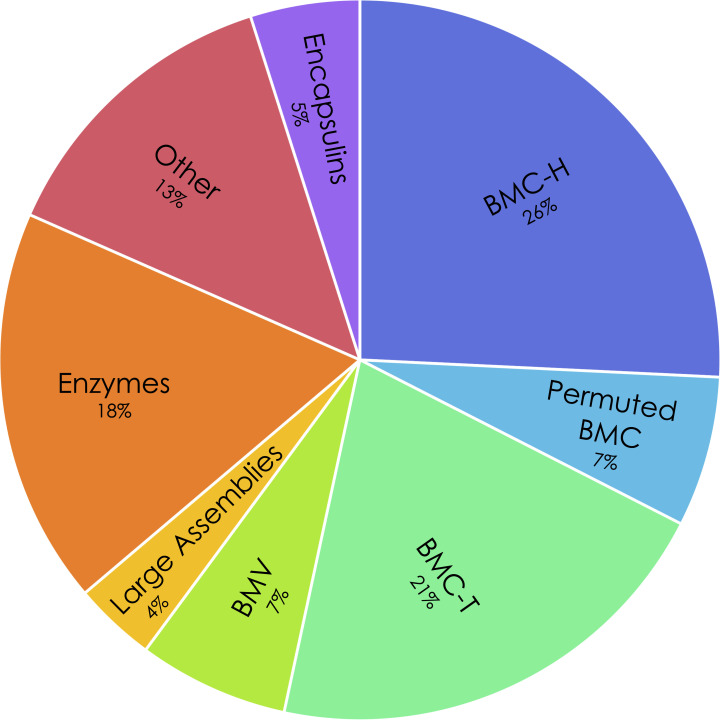
Distribution of protein structure types in the MCPdb. More than 60% of all structures are microcompartment BMC shell proteins (BMC-H, permuted BMC, BMC-T) or pentamers (BMV), with larger icosahedral assemblies comprising 4%, internal enzymes comprising 18% and other microcompartment associated proteins comprising 13%. Encapsulin structures make up the remaining 5% of the MCPdb.

**Fig 5:**
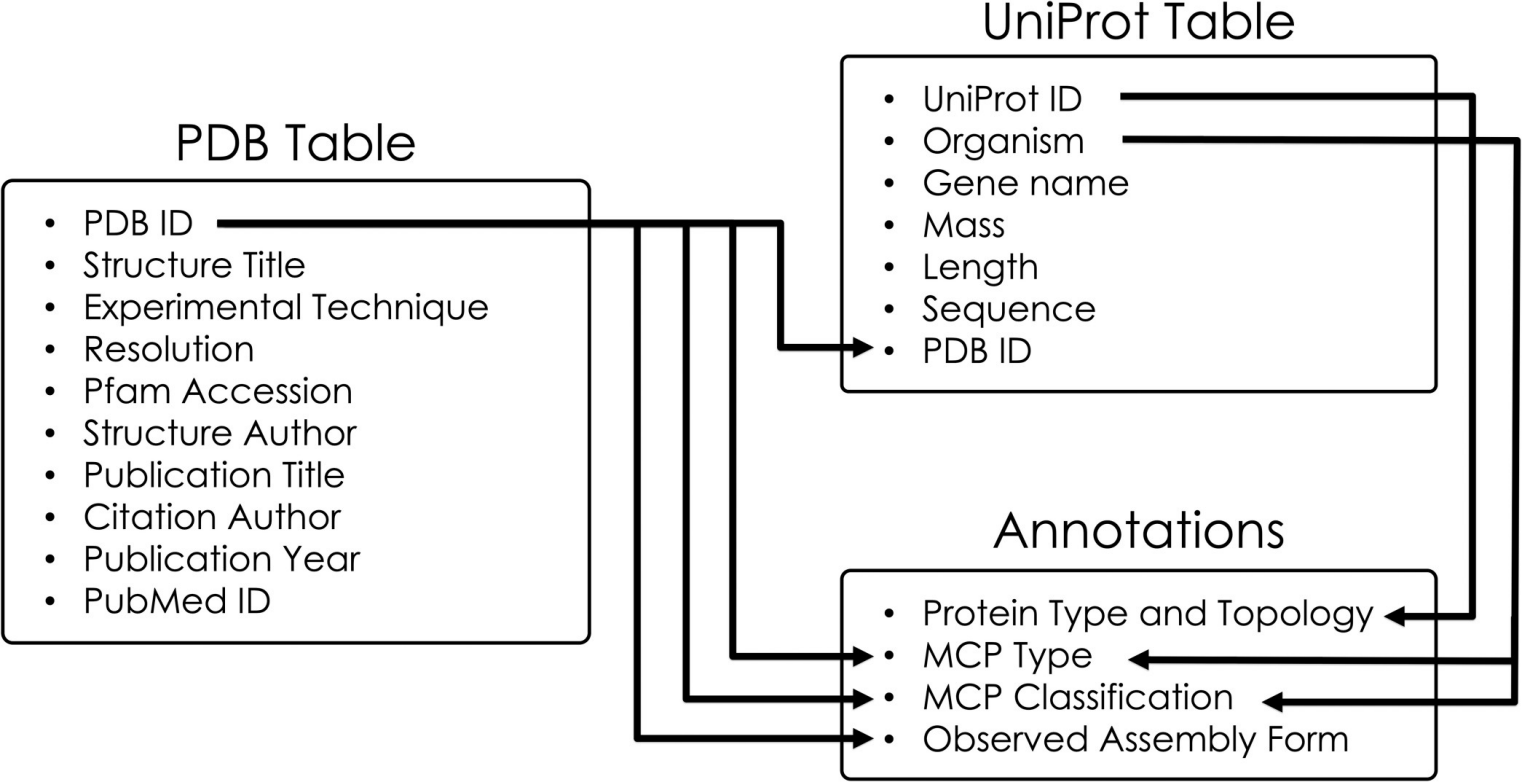
Data sources and annotations for entries in the MCPdb. Key structural information from the PDB as well as associated protein information from UniProt are used to describe each entry. The PDB is the primary link to UniProt IDs. The PDB data file provides information about the Observed Assembly Form for the protein, and UniProt provides information from which the protein topology (e.g. circular permutations and domain duplications) can be discerned. These data sources and the literature are used to annotate the MCP functional type and subclassification.

MCPdb is built by extracting relevant data from the Protein Data Bank [47] and UniProt [48]. We compiled a list of 163 bacterial microcompartment and encapsulin-related structures. A preliminary list of relevant structures was obtained using keyword searches through the PDB web server (https://www.rcsb.org/). An initial search of the term *microcompartment* yielded 112 structures that required manual validation and verification, resulting in a total of 98 structures related to MCPs. In order to curate a more comprehensive list, we performed searches of structures using the amino acid sequences of representative BMC-H, BMC-T, BMV and permuted BMC structures. With the addition of several structures from the unrelated encapsulins, MCPdb presently consists of 163 structures (Fig 4). We performed an HTML-based query to collect relevant information including structure resolution, deposition authors and citations (Fig 5). After generating a master list of PDB IDs, we curated their corresponding amino acid sequences obtained from UniProt. A total of 91 unique UniProt IDs gives rise to the 163 separate PDB structures.

**Fig 6:**
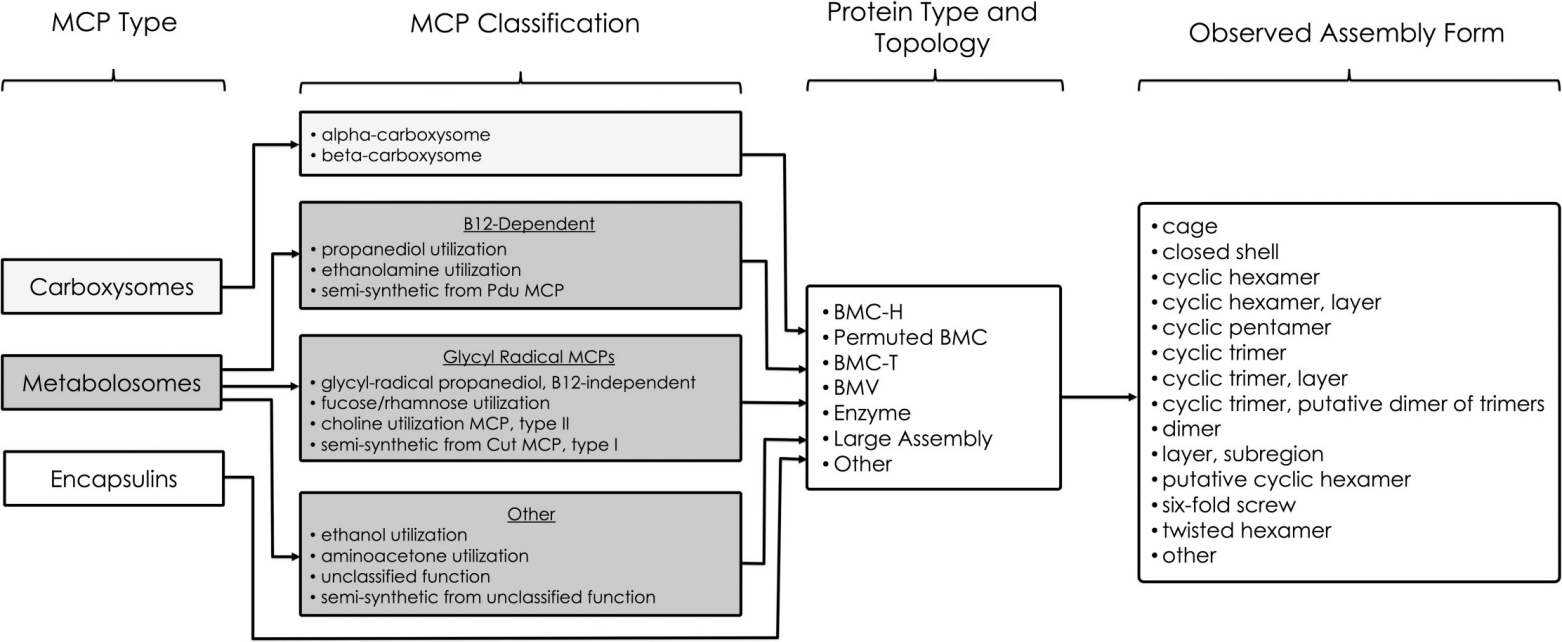
MCPdb entry annotations. MCP Type indicates the broad metabolic category. MCP Classification further distinguishes between the different metabolic subtypes of carboxysomes and metabolosomes. Protein Type and Topology describe properties inherent to the protein tertiary structure. Lastly, Observed Assembly Form describes protein quaternary characteristics of the experimentally described structure.

Upon collecting all relevant data from the PDB and UniProt, we assigned a series of classifications and annotations to each structure. While individual PDB IDs were used as a key for pertinent structural information, the UniProt IDs were used to provide additional protein details (Fig 5). Each structure in the database has been assigned an *MCP Type*, *MCP Classification*, *Protein Type and Topology*, and *Observed Assembly Form* (Fig 6). *MCP Type* broadly categorizes each structure as a carboxysome, a metabolosome or an encapsulin, while *MCP Classification* provides more details about the microcompartment based on its metabolic function, distinguishing between alpha/beta carboxysomes, and the different metabolosome types including the propanediol utilization MCP, ethanolamine utilization MCP and others. We likewise categorized each structure by intrinsic characteristics including *Protein Type and Topology* and *Observed Assembly Form*. While the *Protein Type and Topology* are inherent and describe the type of protein for a given structure (*i*.*e*. BMC-H, BMC-T, BMV, etc.), the *Observed Assembly Form* describes the experimental crystal packing (in some cases) and presumptive quaternary architectures.

**Fig 7:**
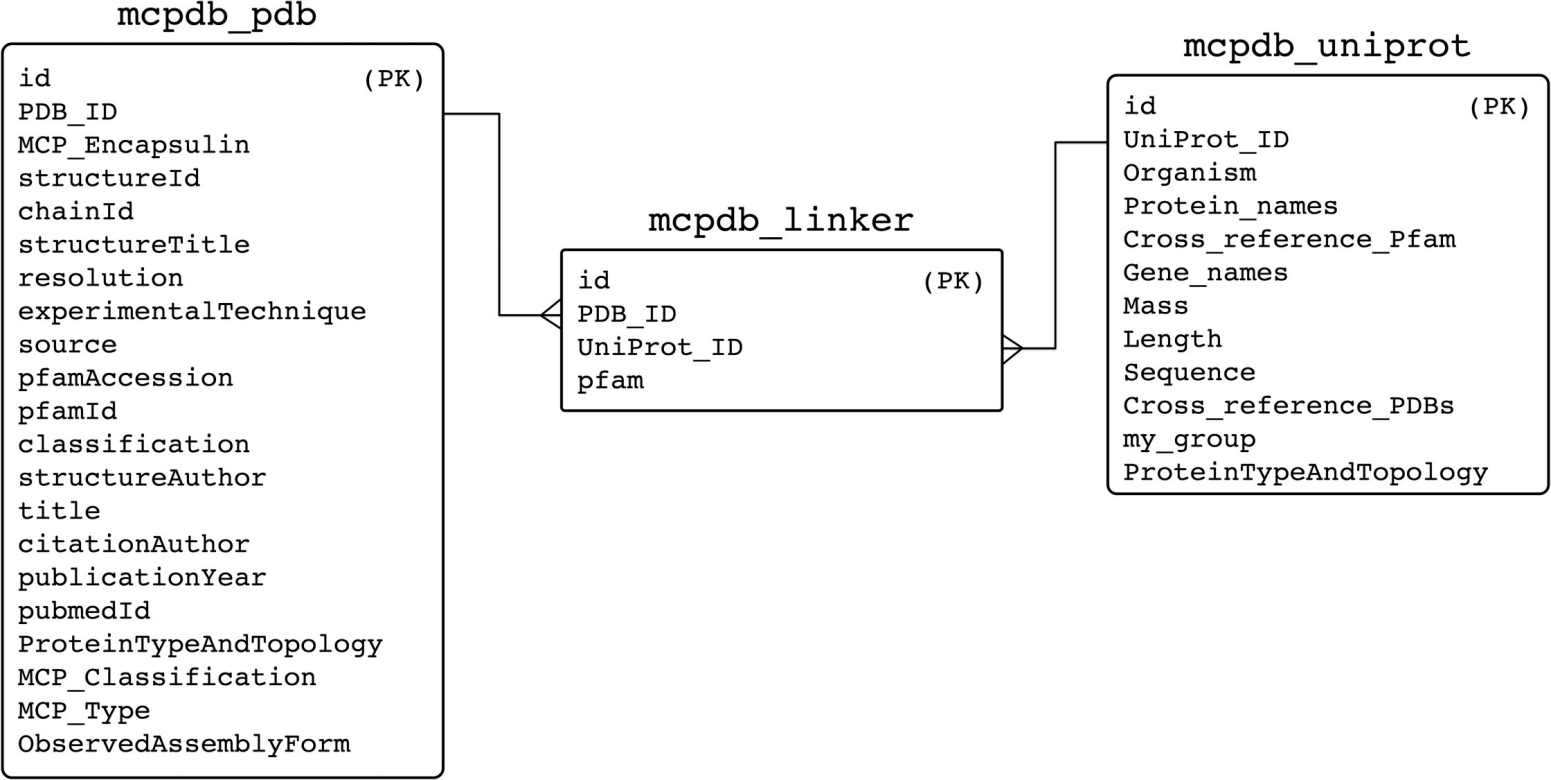
Entity relationship diagram of the MCPdb as a MySQL database. Boxes show the primary data sources and the linker table used to join the tables in the queries. Primary keys (PK) have been identified.

SQL tables were created to link PDB IDs, UniProt IDs and annotations. In order to construct our database, we utilized a Linux server running Ubuntu 20.04 LTS and MySQL version 5.7. CSV files of the PDB data, UniProt data and annotations were converted into SQL tables with the construction of a linker table to join the tables in the query and to establish the one-to-many relationships between PDB IDs and UniProt IDs (Fig 7). One UniProt can be associated with numerous PDBs (i.e. if the same protein has been structurally characterized in the context of multiple experiments) and one PDB can be associated with numerous UniProts (i.e. if the structure characterized is comprised of proteins of more than one identity). We then generated a series of PHP scripts to query the data and populate our website content. Structures on MCPdb are organized and called by their four-character PDB ID.

### File curation and preparation

In order to construct a centralized microcompartment database, we extracted and compiled relevant files including PDBs, biological assemblies, and FASTA amino acid sequence files with the goal of providing these files to the end user. We also sought to provide users with numerous modes of interacting with each structure. To appeal to experts and novices alike, we incorporated: (1) an interactive 3D viewer for rapid structure interrogation, (2) ready-to-use PyMOL graphics sessions for streamlined figure preparation, and (3) images for quickly viewing and interpreting structures while browsing the database.

**Fig 8:**
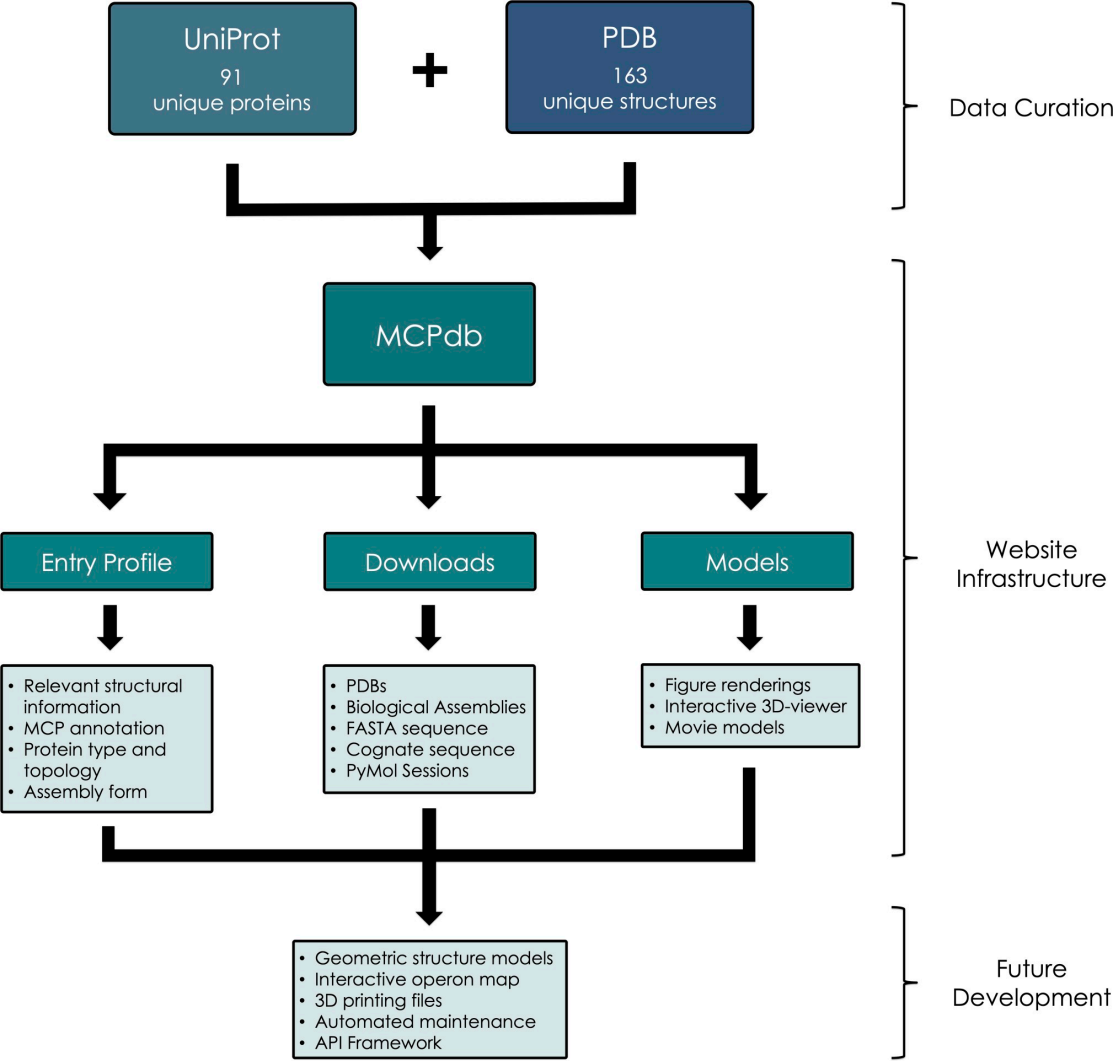
Flow chart depicting data curation and generation, website infrastructure and goals for future development.

All files are housed on our permanent institutional web server using the PDB ID as the primary identifier. With the master list of 163 PDB IDs, we utilized a wget command to pull atomic coordinates of all structures in the form of.pdb and.cif files onto our Linux server, which are available to our users as downloads. We were also able to retrieve nearly 60% of the correctly named and trimmed biological assemblies using the program PISA [49]. The biological assemblies that were generated by PISA and migrated to our server were validated for accuracy. The remaining structures whose biological assemblies could not be successfully generated with PISA required manual intervention; the need for this step highlights one of the key utilities of the database. Because a structure file may contain multiple sets of coordinates for the same set of atoms (that are distinguished by unique models), we used PyMOL to create new.pdb files in which we assigned a unique chain ID to each chain so that these biological assemblies can be easily loaded, free of multiple objects and multiple states. These validated biological assemblies have been cleaned to exclude most small molecules judged to not reflect biological function (e.g. crystallization buffer molecules, etc.). In a few select cases, the natural biological assembly form of a BMC protein remains uncertain (some BMC-T trimers tend to occur in structural studies in the form of two stacked disks). In those cases, users can access alternate assembly forms. MCPdb also provides relevant sequence information in the form of.fasta and.txt files. FASTA-formatted sequences for each structure were retrieved from the PDB; these reflect the actual sequence of the experimentally characterized protein, which can include mutations and the addition of protein purification tags. The native, unmodified protein amino acid sequences (.txt) are extracted from the UniProt data using a PHP query. These files are also available as downloads (Fig 8).

We incorporated an interactive 3D viewer that enables users to dynamically engage with most of the MCPdb structures without the need to download additional molecular visualization software (Fig 8). The mutation position imaging toolbox (MuPIT) is a browser-based visualization application originally designed for novice structure investigators [50]. By integrating this unmodified software into our database, we provide users the opportunity to quickly visualize structures of interests on desktop and mobile-based browsers. Users may view ribbon, line and stick models of each structure. About 8% of structures were too large for the interactive viewer (some contain as many as 540 protein chains), in which cases the server offers movies (created in PyMOL) that dynamically change views and toggle through ribbon and surface renderings of the structure of interest. The short movies of these structures are played in the browser and can also be downloaded and saved locally.

Additionally, we provide ready-to-use PyMOL session files (.pse) as optional downloads. These are functional even for the largest of the structures. After curating and manually validating our library of biological assemblies, we prepared a series of PyMOL sessions (Fig 8). For each structure, we provide users with a cartoon and surface representation of each structure. Structures are colored such that users can rapidly distinguish between multiple polypeptide chains. Surface representations are semi-transparent for easy viewing. We have also pre-loaded short movies so that users are immediately presented with a rotating view of the selected structure upon launching the PyMOL session. Rendering surface representations of large structures, including cages and closed shells, is computationally taxing and can crash PyMOL under some computer user configurations. To overcome these challenges, we employed various lesser-known PyMOL strategies. By reducing the surface quality and altering the Gaussian resolution option in the Fourier filtering representation prior to generating isosurface maps, we were able to create surface representations, even for the largest structures, which are visually informative while requiring significantly reduced computing power. Our uniform PyMOL sessions create an effortless way for novice PyMOL users to interact with each of the 163 structures and a simple way of preparing accurate and illustrative figures. Lastly, we generated a series of three figure-ready images (.pngs) for users to scroll through as they are browsing a structure on MCPdb (Fig 8). Based on specifically crafted PyMOL sessions, we exported a series of views as.pngs and added these as sliders to each entry in the MCPdb. We additionally created and included N to C-terminus color-ramped cartoon diagrams of the asymmetric unit of each structure in the image slider.

### Website construction

Following database curation, we generated a user-friendly browser interface. The MCPdb infrastructure was created using WordPress, HTML, CSS and JavaScript. We utilized the WordPress graphical user interface (GUI) to build the landing page and accessory information pages. We used HTML, CSS and JavaScript to generate a template page that displays select information for each structure. We replicated and auto-populated data fields in this template for each of the 163 structures using a PHP script. We also created a series of queries to provide our users with seamless and intuitive search features. Infrastructure for simple searches based on key words and more complex filtering searches were also created using PHP.

## Results and discussion

### Database description

**Fig 9:**
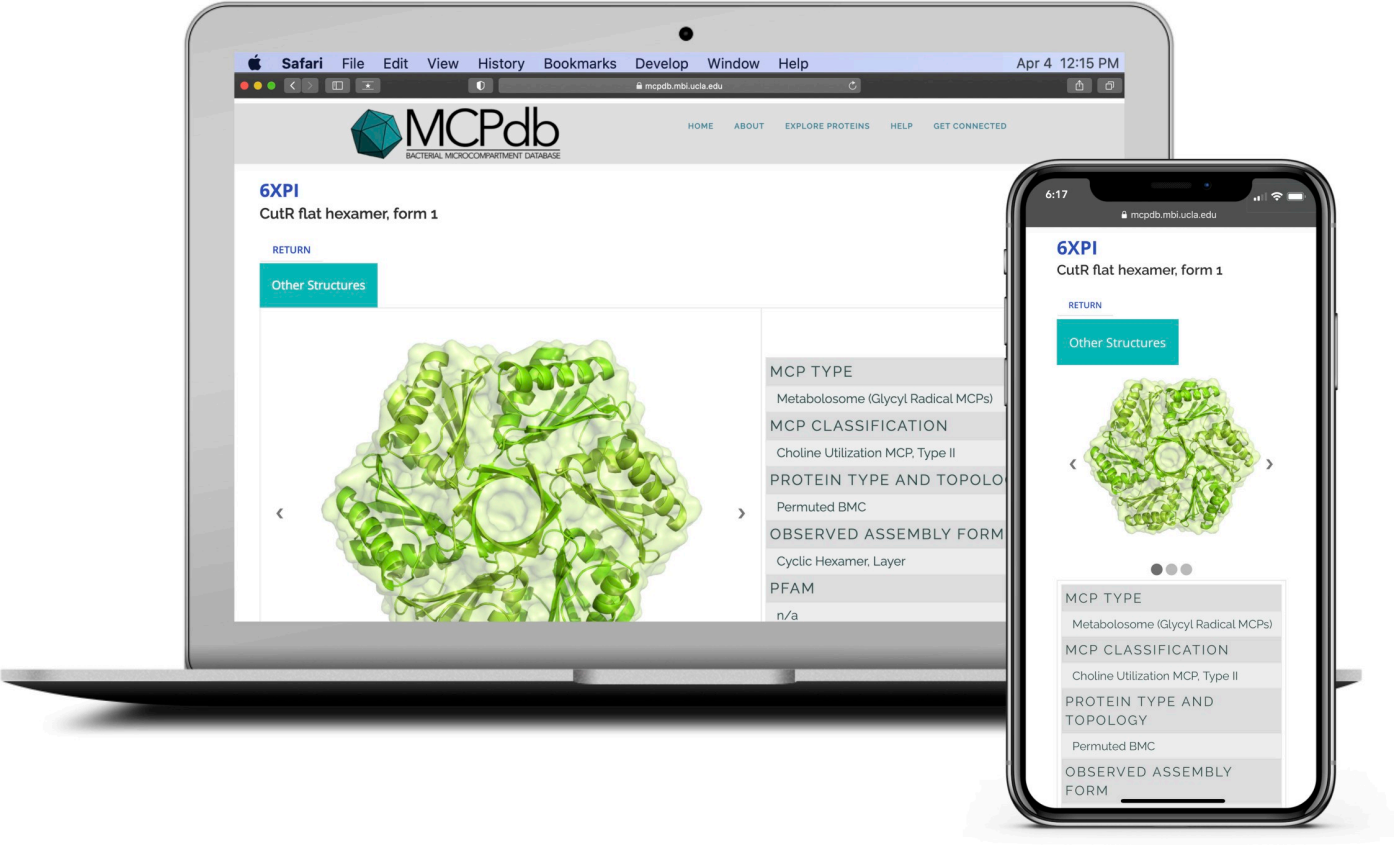
Screenshot and example of a structure profile on the MCPdb interface. MCPdb has been optimized for use on desktops, tablets and mobile devices.

MCPdb is available at https://mcpdb.mbi.ucla.edu, a permanent institutional URL managed by the UCLA-DOE Institute for Genomics and Proteomics. MCPdb was created in order to compile and consolidate structures related to MCPs, and encapsulin structures that are known at this time. More importantly, MCPdb was designed to provide users with readily available structural and biophysical annotations as well as validated biological assemblies. The current version of our database pulls together 163 structures from the PDB (comprising 91 unique UniProts) and a collection of curated files and utilities that are available for in-browser viewing and download (Fig 8). Downloads include PDB files, biological assembly structures (pdb format), files for biological and experimental protein sequences, and ready-to-use PyMOL session files. Users can view rendered images of each structure or interact with them in 3D within the browser. In alignment with our philosophy of introducing new users to the field, MCPdb is freely available and optimized for accessibility on desktops, tablets and mobile devices (Fig 9).

### Web interface

MCPdb provides a simple and interactive framework for users to explore bacterial microcompartments and encapsulins. Upon navigating to the home page, users are presented with a brief database description and provided with links that navigate to a summary page, search page and a quick-start guide. As users explore MCPdb, they are introduced to high-level information about MCPs and their characteristic shell proteins. As they navigate to an individual entry page, users are provided with images of the structure and relevant annotations including *MCP Type*, *MCP Classification*, *Protein Type and Topology* and *Observed Assembly Form* (Fig 9). Users can scroll down for additional information related to the structure and authorship, they can download validated structure files and ready-made PyMOL sessions or they can view the structure in 3D within their browser. We additionally provide a *Get Connected* page to allow users to request assistance and provide feedback.

### Comparison to other databases

The MCPdb combines data available from other sources, including the PDB [30] and UniProt [31], with curation and substantial post-processing. The various curation and postprocessing protocols add considerable value compared to currently available data repositories. Presentation of correct biological assembly states is often a challenge for structures obtained by crystallographic methods, and as noted above this is a critical aspect of interrogating MCP structure and function. Vital information, and search capacity, is also provided concerning metabolic function types and unique topological features in the BMC protein family; these features relate to functional differences in their assembly and their roles in molecular transport. There are some parallels between MCPs and viral capsids, and indeed the need for a database that curates biological assembly forms for viral capsids was recognized some years ago with the development of the VIPERdb database [51]. Similarly, other specialized databases that systematically collect, annotate and process structures using expert curation, including KLIFS (Kinase–Ligand Interaction Fingerprints and Structures), have provided researchers with valuable feature-rich resources [52]. The MCPdb answers an analogous need for bacterial microcompartments.

Curation has been applied to remove complicating accessory data (e.g. bound buffer molecules, conflicting polypeptide chain names, etc.), which might otherwise confuse non-expert users. The integration with multiple modes of visualization, tailored where necessary according to size, will facilitate the graphical display and dissemination of information on these special biological systems. Attention has been given to providing simple methods of display to serve the broadest community of users.

## Conclusions and future prospects

The MCPdb currently houses 163 microcompartment protein and encapsulin-related structures. Access to validated biological structures as well as structural and biophysical annotations is necessary for well-informed scientific investigation surrounding MCPs. As a relatively new field, the structural biology of MCPs is an area of growing scientific and bioengineering interest [53–64]. Not only a tool for experts in the field, the MCPdb provides novices and young students the opportunity to learn about and explore bacterial MCPs. The exceptional biological role of MCPs as protein-based organelles makes them an attractive subject for young scientists, as they challenge the textbook paradigm that eukaryotic cells possess mechanistically complex subcellular organelles while bacterial cells do not.

As the body of structural data on MCPs grows, increased automation will be required to keep the database current. Ongoing developments will involve methods to periodically survey the PDB for new microcompartment and encapsulin-related structures, and their associated data files. Additionally, further efforts will expand the types of information and utilities available on the database. Subsequent versions will introduce an interactive operon map for exploring the operon structure and genomic context of BMC shell proteins and their associated encapsulated enzymes. We are also working to provide users with further geometric representations of the structures, electrostatic potentials, pore properties and graphics files for 3D printing, as well as an API framework to extend the functionality of MCPdb for future users. These capabilities will further facilitate access to the field of MCPs for basic and applied research.
